# Carbon footprint of atrial fibrillation catheter ablation

**DOI:** 10.1093/europace/euac160

**Published:** 2022-09-15

**Authors:** Geoffroy Ditac, Pierre-Jean Cottinet, Minh Quyen Le, Daniel Grinberg, Josselin Duchateau, Kévin Gardey, Arnaud Dulac, Antoine Delinière, Christelle Haddad, Julie Boussuge-Roze, Frédéric Sacher, Pierre Jaïs, Philippe Chevalier, Francis Bessière

**Affiliations:** Department of Electrophysiology, Hôpital Cardiologique Louis Pradel, Hospices Civils de Lyon, 28 avenue du Doyen Lepine, 69500 Bron, France; INSA-Lyon, LGEF, Université de Lyon, 20 Av. Albert Einstein, 69100 Villeurbanne, France; INSA-Lyon, LGEF, Université de Lyon, 20 Av. Albert Einstein, 69100 Villeurbanne, France; Department of Cardiac Surgery, Hôpital Cardiologique Louis Pradel, Hospices Civils de Lyon, 28 avenue du Doyen Lepine, 69500 Bron, France; Université Claude Bernard Lyon 1, Faculté de Médecine Lyon Est, 8 avenue Rockefeller, 69003 Lyon, France; Department of electrophysiology, CHU Bordeaux, Université de Bordeaux, IHU LIRYC, Av. du Haut Lévêque, 33600 Pessac, France; Department of Electrophysiology, Hôpital Cardiologique Louis Pradel, Hospices Civils de Lyon, 28 avenue du Doyen Lepine, 69500 Bron, France; Department of Electrophysiology, Hôpital Cardiologique Louis Pradel, Hospices Civils de Lyon, 28 avenue du Doyen Lepine, 69500 Bron, France; Department of Electrophysiology, Hôpital Cardiologique Louis Pradel, Hospices Civils de Lyon, 28 avenue du Doyen Lepine, 69500 Bron, France; Université Claude Bernard Lyon 1, Faculté de Médecine Lyon Est, 8 avenue Rockefeller, 69003 Lyon, France; Department of Electrophysiology, Hôpital Cardiologique Louis Pradel, Hospices Civils de Lyon, 28 avenue du Doyen Lepine, 69500 Bron, France; Department of electrophysiology, CHU Bordeaux, Université de Bordeaux, IHU LIRYC, Av. du Haut Lévêque, 33600 Pessac, France; Department of electrophysiology, CHU Bordeaux, Université de Bordeaux, IHU LIRYC, Av. du Haut Lévêque, 33600 Pessac, France; Department of electrophysiology, CHU Bordeaux, Université de Bordeaux, IHU LIRYC, Av. du Haut Lévêque, 33600 Pessac, France; Department of Electrophysiology, Hôpital Cardiologique Louis Pradel, Hospices Civils de Lyon, 28 avenue du Doyen Lepine, 69500 Bron, France; Université Claude Bernard Lyon 1, Faculté de Médecine Lyon Est, 8 avenue Rockefeller, 69003 Lyon, France; Department of Electrophysiology, Hôpital Cardiologique Louis Pradel, Hospices Civils de Lyon, 28 avenue du Doyen Lepine, 69500 Bron, France; Université Claude Bernard Lyon 1, Faculté de Médecine Lyon Est, 8 avenue Rockefeller, 69003 Lyon, France

**Keywords:** Catheter ablation, Atrial fibrillation, Environment, Carbon footprint, Eco-audit

## Abstract

**Aims:**

Climate change represents the biggest global health threat of the 21st century. Health care system is itself a large contributor to greenhouse gas (GHG) emissions. In cardiology, atrial fibrillation (AF) catheter ablation is an increasing activity using numerous non-reusable materials that could contribute to GHG emission. Determining a detailed carbon footprint analysis of an AF catheter ablation procedure allows the identification of the main polluting sources that give opportunities for reduction of environmental impact. To assess the carbon footprint of AF catheter ablation procedure. To determine priority actions to decrease pollution.

**Methods and results:**

An eco-audit method used to predict the GHG emission of an AF catheter ablation procedure was investigated. Two workstations were considered including surgery and anaesthesia. In the operating room, every waste produced by single-use medical devices, pharmaceutical drugs, and energy consumption during intervention were evaluated. All analyses were limited to the operating room. Thirty procedures were analysed over a period of 8 weeks: 18 pulmonary veins isolation RF ablations, 7 complex RF procedures including PVI, roof and mitral isthmus lines, ethanol infusion of the Marshall vein and cavo tricuspid isthmus line, and 5 pulmonary vein isolation with cryoballoon. The mean emission during AF catheter ablation procedures was 76.9 kg of carbon dioxide equivalent (CO_2_-e). The operating field accounted for 75.4% of the carbon footprint, while only 24.6% for the anaesthesia workstation. On one hand, material production and manufacturing were the most polluting phases of product life cycle which, respectively, represented 71.3% (54.8 kg of CO_2_-e) and 17.0% (13.1 kg of CO_2_-e) of total pollution. On the other hand, transport contributed in 10.6% (8.1 kg of CO_2_-e), while product use resulted in 1.1% (0.9 kg of CO_2_-e) of GHG production. Electrophysiology catheters were demonstrated to be the main contributors of environmental impact with 29.9 kg of CO_2_-e (i.e. 38.8%). Three dimensional mapping system and electrocardiogram patches were accounting for 6.8 kg of CO_2_-e (i.e. 8.8% of total).

**Conclusion:**

AF catheter ablation involves a mean of 76.9 kg of CO_2_-e. With an estimated 600 000 annual worldwide procedures, the environmental impact of AF catheter ablation activity is estimated equal to 125 tons of CO_2_ emission each day. It represents an equivalent of 700 000 km of car ride every day. Electrophysiology catheters and patches are the main contributors of the carbon footprint. The focus must be on reducing, reusing, and recycling these items to limit the impact of AF ablation on the environment. A road map of steps to implement in different time frames is proposed.

## Graphical Abstract

**Graphical Abstract:**
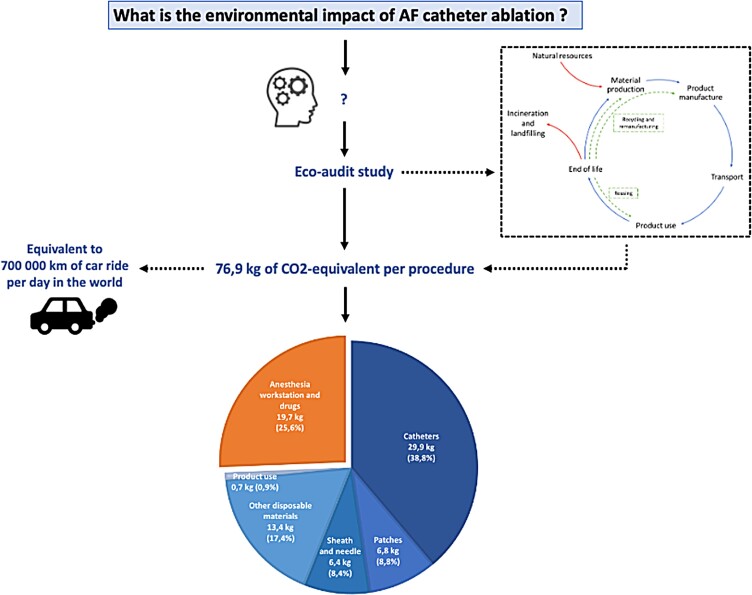

**What’s new?**

- This prospective study is the first to use an eco-audit method to determine the carbon footprint of atrial fibrillation (AF) ablation.
- 76.9 kg of CO_2_-equivalent is emitted during an AF catheter ablation procedure. This is equivalent to 700 000 km of car ride each day in the world.
- Electrophysiology catheters, sheaths, and patches are the main polluting sources, accounting for more than half of the carbon footprint.
- A road map to reduce the impact of AF catheter ablation on the environment is proposed. It includes reducing, recycling, reprocessing, and redesigning of single-use devices.

## Introduction

Global warming is an increasing problem and will be one of the top challenges of the 21st century. It is caused by greenhouse gas (GHG) emission with irremediable consequences on climate and public health. Paradoxically, the healthcare sector is one of the biggest contributors of GHG emission. It is estimated to account for 5% of global GHG emission and up to 10% in the developed countries.^1–3^ Studying the ecological impact of the health care system would help decision-makers to guide policy toward a more sustainable system. Yet, environmental studies in medicine are scarce.

Eco-audit is a powerful tool to determine the GHG emission during the different phases of a product life. It is useful to assess the current situation and identify areas for improvement. This method has recently been exploited in cardiac surgery, determining that a conventional isolated cardiac procedure contributes to global warming in the same way as a 1080 km plane flight.^4^

Cardiac electrophysiology (EP), which requires a lot of sophisticated single-use material (but possibly reusable), is an interesting study case for eco-audit. Atrial fibrillation (AF) catheter ablation was considered for this work, as it is the most performed and standardized procedure in cardiac EP. Based on an estimation of 600 000 AF ablations every year, it represents a procedure every minute, and this amount is rapidly increasing as AF incidence grows worldwide.^5–7^

This work aims at estimating the global and detailed carbon footprint of an AF ablation procedure in order to sensitize physicians and industrials. Our study allows to determine possible priority actions needed to reduce GHG emission caused by EP ablation procedure.

## Methods

### Study design and patient selection

Data were reported from a prospective, observational, single-tertiary centre study (Hôpital cardiologique Louis Pradel—Hospices Civils de Lyon). We considered for the eco-audit every adult (i.e. >18 years old) patient undergoing first or redo AF catheter ablation, with radiofrequency (RF) energy or single shot cryoballoon device. All procedures were performed under general anaesthesia. Procedures were performed by any operator of the department. Data collection consisted of disposable materials, pharmaceutical products (including intravenous drugs and anaesthetic gas), and energy consumption (electricity and gas) used during each intervention. No clinical data related to the patients were collected aside the type of arrhythmia and details regarding material needed for the intervention itself. The procedures were analysed in three different groups: *RF-PVI*, pulmonary vein isolation (PVI) using irrigated tip RF energy; *RF-Marshall*, PVI + roof and mitral isthmus lines + CTI line using irrigated RF energy and ethanol infusion of the Marshall vein; *Cryo-PVI*, PVI with a cryoballoon catheter.

Data collection was performed by a single physician (G.D.) with help of a biomedical expert (P.-J.C.). This study was approved by our institutional ethics committee (Comité d’éthique des Hospices Civils de Lyon).

### Carbon footprint measurement

**Figure 1:**
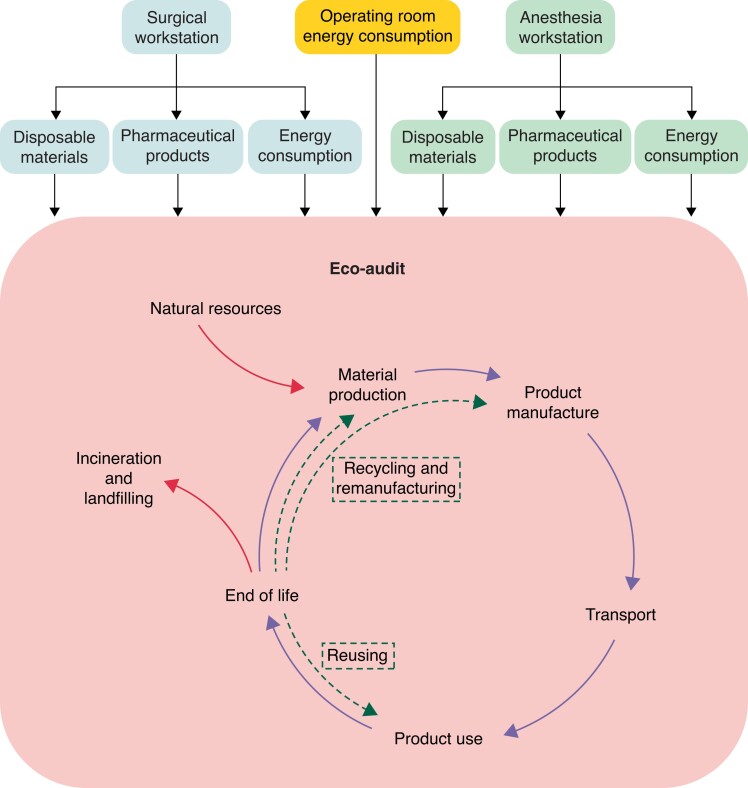
Eco-audit principle used to estimate the energy consumption of surgical and anaesthesia workstations during an atrial fibrillation catheter ablation procedure.

The eco-audit method proposed by Ashby^8^ to quantify environmental GHG emission was used (*Figure 1*). The anaesthesia and the surgical workstations were identified in our analysis. Only information that was taking place in the operating room was considered. Three groups of polluting sources were gathered for each workstation.

- Disposable materials (including catheters, sheath, patches, surgical drapes, syringes, needles, etc.): being weighed and analysed during the period between the patient’s entry and exit of operating room.
- Pharmaceutical products (including anaesthetic gases).
- Energy consumption: Electricity consumption of each equipment (light, airflow, computers, screens, fluoroscopy generator, ventilation machine, etc.) in the operating room was collected. Data related to the surgical platform of our hospital were provided by the administration.

The eco-audit approach is a fast initial assessment of the energy demands or carbon emissions of a product’s life, which is splitted into different phases: material, manufacturing, transportation, use, and disposal. Product use represents the energy consumption (electricity and gas) of the device during its use. For each phase, a CO_2_ production is calculated using different inputs. The main challenge of the study is to determine accurately all the inputs: original substance, production site, shaping process, mode of transportation, duty cycle, disposal route, etc. Most of the information can be found on the label or referring to the CE marking. Consumable analysis and carbon production estimate were made by an independent team of scientists and engineers (LGEF Laboratory at INSA—Université de Lyon).

### Data analyses

Calculations were performed with Ansys Granta EduPack software. The quantification of GHG was expressed as ‘Carbon dioxide equivalent’ (CO_2_-e) calculated using the GWP-100 (Global Warming Potential on a 100-years period). Differences between RF-PVI, RF-Marshall and Cryo-PVI regarding continuous variables were reported as mean ± SD, and discrete variables were reported as proportions (percentage). Statistical analyses were performed using R version 3.6.3 (R Foundation for Statistical Computing, Vienna, Austria).

## Results

### Overview

Over an 8-week period, a total of 30 non-consecutive AF ablation procedures were evaluated: 18 RF-PVI, 7 RF-Marshall, and 5 Cryo-PVI. The mean duration of procedures (from patient entrance to exit of operating room) was 140 ± 37 min.

**Figure 2:**
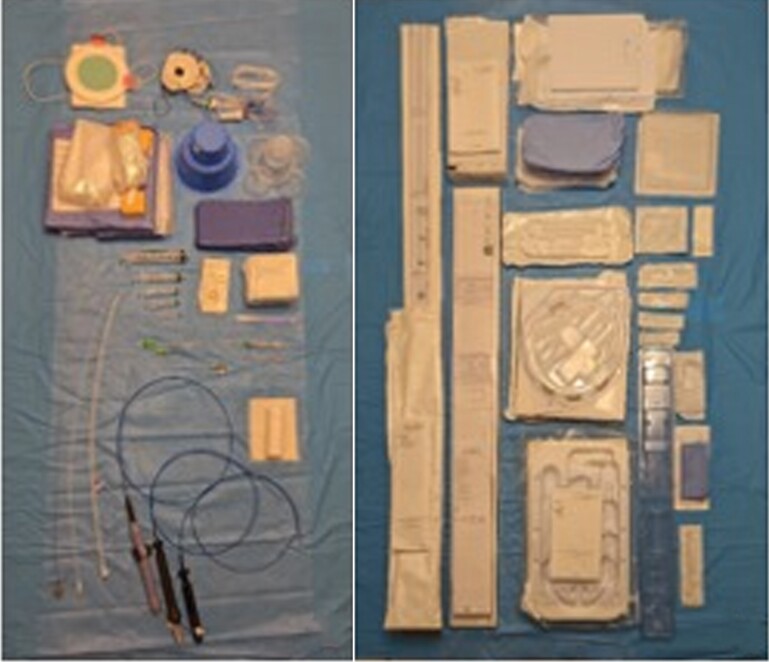
Material for a single atrial fibrillation catheter ablation procedure and its packaging.

**Figure 3:**
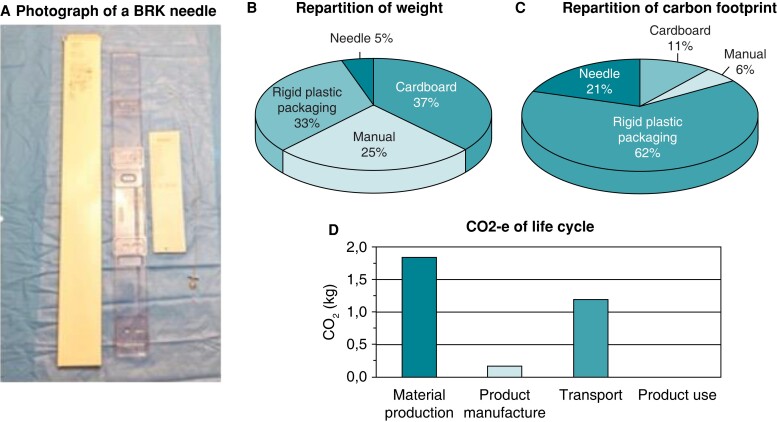
Example of eco-audit analysis on a BRK transseptal needle (BRK-1, St. Jude Medical, Inc., St. Paul, MN, USA) (part A); the repartition of weight (part B) is compared with the repartition of carbon footprint (part C) and carbon dioxide-equivalent (CO_2_-e) life cylce (part D).

Every material used during ablation procedure was studied to obtain its carbon footprint based on the following steps: material, manufacture, transportation, and product use (*Figure 1*). We evaluated composition of each device together with its packaging, as depicted *Figure 2*. As expected, polymers were the most used, representing around 88% of the total weight. *Figure 3* shows example of results obtained with a transseptal needle (BRK-1, St. Jude Medical, Inc., St. Paul, MN, USA).

### Description of procedures

**Figure 4:**
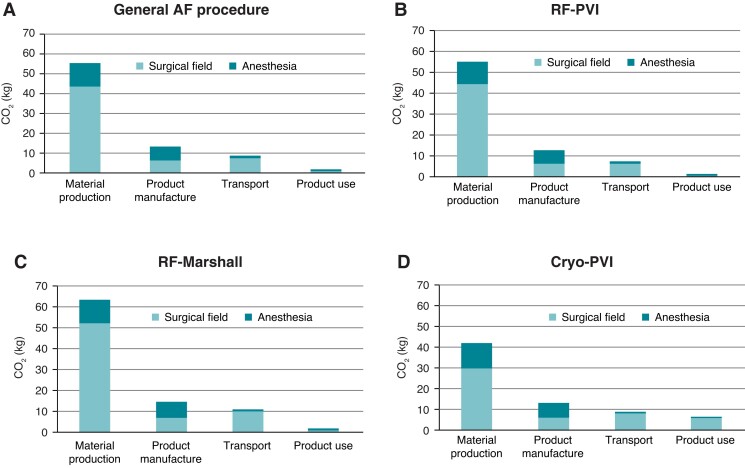
Life cycle analysis for each workstation. All results are expressed in kg of carbon dioxide equivalent. AF, atrial fibrillation; RF-PVI, pulmonary vein isolation (PVI) using irrigated tip RF energy; RF-Marshall, PVI + roof and mitral isthmus lines + CTI line using irrigated RF energy and ethanol infusion of the Marshall vein; Cryo-PVI, PVI with a cryoballoon catheter.

The mean global GHG emission during an AF catheter ablation procedure was 76.9 kg of CO_2_-e. This result does not consider recycling or reprocessing. The GHG emissions generated by the two workstations (surgical and anaesthesia fields) are presented in *Figure**4A* and *E*. The surgical field, with 58.0 kg of CO_2_-e (∼75.4%), gave raise to higher carbon footprint compared with anaesthesia. Material production was the most polluting phase of product life cycle, indicating 71.3% (54.8 kg of CO_2_-e) of total pollution. Product manufacture represented 17.0% (13.1 kg of CO_2_-e) and transport 10.6% (8.1 kg of CO_2_-e) of pollution. Product use only accounted for 1.1% (0.9 kg of CO_2_-e) of GHG emission.

Comparison of the three different groups is shown in *Figure 4B–D*. RF-Marshall procedures were the most voracious consumer of GHG, with a total of 87.9 kg of CO_2_-e per procedure. RF-PVI and Cryo-PVI led to lower energy consumption, with 75.8 and 71.2 kg of CO_2_-e, respectively. While the main contributor of carbon footprint is material production, we observed an increase of pollution due to product use with cryoballoon (6.3 kg of CO_2_-e) because of nitrogen.

### Description of materials

**Figure 5:**
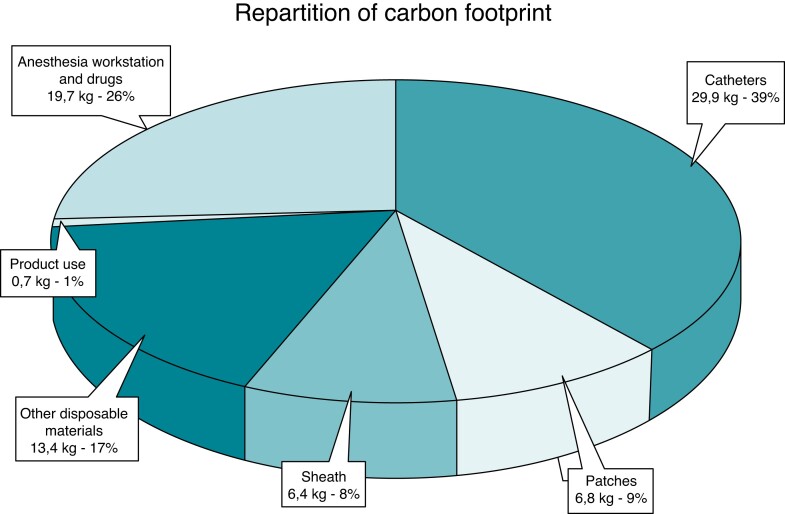
Carbon dioxide footprint estimated distribution for a single atrial fibrillation catheter ablation procedure.

**Figure 6:**
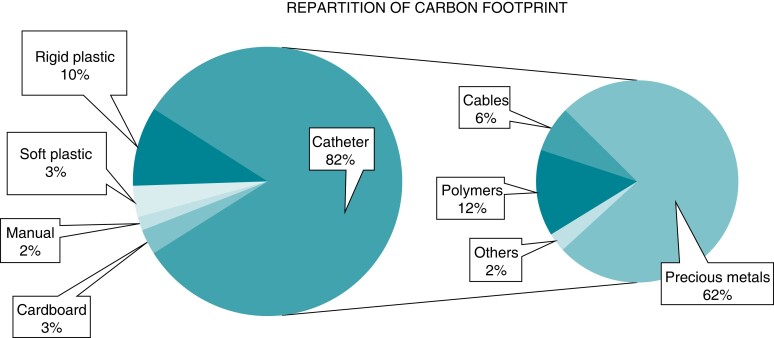
Greenhouse gas distribution of different catheter’s components.

When observing in detail the polluting sources (*Figure 5*), EP catheters appeared to be the main contributor of CO_2_ production (38.8%; 29.9 kg of CO_2_-e). The carbon footprint of each catheter was evaluated between 8.1 and 15.1 kg of CO_2_-e, in which the most consuming were the RF ablation catheter (SmartTouch SF®, Biosense Webster, Inc., CA, USA) and the cryoballoon (FlexCath Advance™, Arctic Front Advance Pro™ and Achieve™, Medtronic, Inc. MN, USA). Analysis of catheter components showed that electrode, containing precious metals, was the most polluting part (*Figure 6*).

Transseptal needle (BRK-1, St. Jude Medical, Inc., St. Paul, MN, USA) was contributing for 3.2 kg of CO_2_-e. Standard sheath (Swartz™ SL0™ sheath and dilator, St. Jude Medical, Inc., St. Paul, MN, USA) was responsible for a 1.2 kg of CO_2_-e, while 6.6 kg for a steerable sheath (Agilis™ NxT steerable introducer, St. Jude Medical, Inc., St. Paul, MN, USA).

Interestingly, all patches used (i.e. carto; electrocardiogram; RF) were the second cause of pollution with an 8.8% ratio (6.8 kg of CO_2_-e). For a single use, defibrillation patches and mapping patches were estimated to produce 1.1 and 3.0 kg of CO_2_-e, respectively. Cables that come with patches greatly contributed to the carbon footprint.

*Table 1* summarizes the most polluting EP materials.

**Table 1:** Carbon footprint of materials used in AF catheter ablation procedure

Disposable material	Estimated carbon footprint (kg of CO_2_-e)
Quadripolar steerable catheter (Inquiry™, St. Jude Medical, Inc., St. Paul, MN, USA)	8.1
Decapolar steerable catheter (Inquiry™, St Jude Medical, Inc., St. Paul, MN, USA)	8.1
High density mapping catheter (Pentaray ®, Biosense Webster, Inc., CA, USA)	10.3
RF ablation irrigated catheter (SmartTouch SF®, Biosense Webster, Inc., CA, USA)	11.8
Cryoballoon system (FlexCath Advance ™, Arctic Front Advance Pro™ and Achieve™, Medtronic, Inc. MN, USA)	15.1
Standard sheath (Swartz™ SL0™, St Jude Medical, Inc., St. Paul, MN, USA)	1.2
Steerable sheath (Agilis™ NxT, St Jude Medical, Inc., St. Paul, MN, USA)	6.6
Transseptal needle (BRK-1, St. St Jude Medical, Inc., St. Paul, MN, USA)	3.2
ECG monitoring electrodes (Ambu® BlueSensor, Ambu A/S, Copenhagen, Denmark)	2.5
RF return electrode (Valleylab™ PolyHesive™ Corded Patient Return Electrodes, Medtronic, Inc. MN, USA)	0.8
Defibrillation patches (Pro-padz®, Zoll Medical Corporation, Chelmsford, MA, USA)	1.1
3D mapping system patches (Carto® 3 system external reference patches, Biosense Webster, Inc., CA, USA)	3.0

CO2-e, carbon dioxide equivalent.

## Discussion

While interventional cardiac EP activity is growing fast, most of the material required for ablation is single use. Understanding the carbon footprint of consumables used in health care system can help to limit the impact on the environment, which is essentially based on optimization of material use. To the best of our knowledge, this study is the first to focus on the field of interventional cardiology and to describe the global carbon footprint of AF catheter ablation.

### Eco-audit and previous studies

In a study with a similar methodology, Grinberg *et al*.^4^ found that a conventional cardiac surgery was responsible for 124.3 kg of CO_2_-e. Thiel *et al*.^9^ published that a hysterectomy produced 424 kg of CO_2_-e. Morris *et al*.^10^ found that a cataract surgery represented 182 kg of CO_2_-e. These large discrepancies are explained because of different methodologies, showing the lack of consensus on adequate method to estimate the carbon footprint. Group of experts and clear guidelines are needed to standardize this new field of research.

### Carbon footprint of AF ablation: overview

A single AF ablation was responsible for a mean of 76.9 kg of CO_2_-e, i.e. equivalent to 420 km of car ride, considering a 180 g/km CO_2_ emission. Assuming an annual worldwide number of 1115550 cardiac catheter ablation in 2019 (pre Covid area) with a 5.9% annual growth^6^ and a 50% share for AF ablation,^7^ it is about 600 000 procedures that are performed each year worldwide. Accordingly, the environmental impact of AF catheter ablation activity is estimated equal to 125 tons of CO_2_ emission each day. Considering our local protocol for performing AF ablation, this is equivalent to a daily car ride of 700 000 km.

In our study, we found complex procedures to be more polluting. This finding was expected as these procedures require more time and materials. We also found that PVI with cryoballoon produced less CO_2_-e than RF. This trend is mainly explained by differences between the catheters. However, these results should be taken with caution as the purpose of our study was not to detect a difference of carbon footprint between procedures.

As one might expect, disposable medical products were the main polluting source. Material production and manufacturing were responsible for nearly 90% of the environmental impact of AF catheter ablation while transport and product use represent a small part of the total. Hence, priority should be given to the optimization of the production process including conception and lines. At the contrary, improvements of transportation and energy consumption during procedure may only lead to small changes on the total environmental impact.

### Main targets: catheter, patches, and packaging

The carbon footprint is mainly linked to the EP material, with catheters, sheaths, and patches representing more than half of the pollution (*Figure 5*). With their sophisticated technology, catheters are essential tools for electrophysiologists. In the meantime, they are the biggest contributor to the carbon footprint of EP procedures with a mean of 29.9 kg of CO_2_-e per procedure (∼38.8%).

Carbon footprint of patches was surprisingly high and their impact on GHG is usually underestimated. Indeed, despite their extremely light weight (only few grams), they participate significantly to CO_2_ emission (∼8.8%). Their impact is explained by the copper contained in the wires and magnets that allow the mapping system to work.

All these disposable products come with their important packaging (*Figure 2*), which is usually ignored in most of study involving environmental impact. Packaging makes a huge impact on the planet with countless resources used in the creation of products often designed to be discarded. Additionally, a large portion of that packaging used to protect medical tools and drugs is manufactured from petrol-based plastic and is never recycled. For instance, in the transseptal needle (BRK-1 transseptal needle, St. Jude Medical, Inc., St. Paul, MN, USA) pack, the needle itself represents only 5% of the weight, which leads to 21% of the carbon footprint, while packaging weight represents 33% with a carbon footprint ratio of 62% (*Figure 3*). Therefore, most of the product’s weight comes from the packaging. Every packaging additional weight has its importance for transportation and needs to be considered as a significant part in the carbon footprint of the device (needle and *Figure 3*).

### Road map to decrease carbon footprint of atrial fibrillation catheter ablation

**Figure 7:**
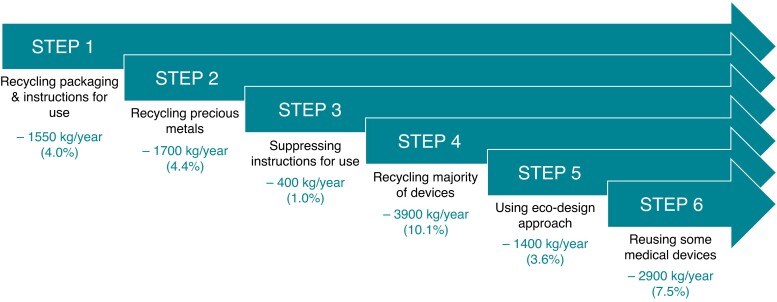
Illustration of a roadmap leading to a decrease in carbon dioxide for a centre performing 500 atrial fibrillation catheter ablations per year. All results are expressed in kg of carbon dioxide equivalent.

According to the above, the devices used to perform AF ablation have a significant impact on the carbon footprint of the procedure. From a medico-ecological point of view, it is therefore necessary to propose action plans to minimize their environmental impact. Here is a road map with several proposals that can be implemented in the short, medium, and long term. For each solution, a costing in terms of CO_2_ reduction has been assessed, based on different hypotheses available in the literatures.^11–13^ The proposed approach refers to the 3Rs principle: reduce, reuse, and recycle.^14–16^*Figure 7* summarizes actions that should result in significantly decreased carbon impact.

### STEP 0. Sensitizing physicians

A recent European survey showed that the EP community is increasingly concerned about the environment.^17^ Physicians have their role to play to reduce the carbon footprint of a procedure. Therefore, in addition to the economic cost, physicians should consider the ecological impact of the materials. As an example, in our study, a steerable sheath produces five times more CO_2_-e than a standard one (*Table 1*). Defibrillator patches also have an important ecological impact and should thus only be used for procedures that absolutely require their presence. The number and complexity of catheters has to be considered. Their number should therefore be limited to what is strictly necessary, and the use of multi-electrode catheters should be restricted to complex cases where substrate mapping is a game changer.

In a more general way, graphical indicators of carbon footprint displayed on every product label would help in changing behaviours in the operating room. An obligation for carbon footprint indicators on every product will certainly have an impact on decisions making process of hospital administrations and physicians within the next decade.

In addition, a significant number of patients require redo procedures, which increase the environmental impact of AF catheter ablation. The subsequent pollution is a new argument for improving single procedure success rate.

### STEP 1. Recycling packaging and instruction for use

The first measure would be to generalize recycling of packaging and instructions in hospitals. Plastic wastes are made with polymers which, if collected before being soiled by the surgical procedures, can be recycled with treatment lines that already exist in most countries.^18,19^ Instruction manual and cardboard packaging can also be easily recycled. According to our estimation, recycling of these materials could reduce carbon footprint by 3.1 kg of CO_2_-e per procedure. This value considers the impact of the recycling phase for different materials.

These recycling measures should also be supported by an eco-design approach that includes reduction of packaging and use of recyclable resources when available.

### STEP 2. Recycling precious metal from catheters

As material production remains the most polluting phase of medical devices life (*Figure 4*), a particular attention must be paid to this phase.

As illustrated in *Figure 6*, the majority of carbon footprint in catheters comes from the use of precious metals, like platinum and gold, whose extraction is highly polluting. These metals could thus be separated from the other materials from the catheter, to be recycled and reused for other industrial purposes. Fritz *et al*.^20^ pointed out that recycling metal instead of extracting allows to save ninefold in energy consumption. In our catheter devices, recycling of these metals could result in a reduction of 3.4 kg of CO_2_-e for each procedure, i.e. equivalent to ∼5% of total GHG emission.

### STEP 3. Suppressing instruction for use

Better than recycling is the reduction or elimination of unnecessary elements. An obvious example is the instruction leaflet in paper format. These documents are required for the labelling process but totally unnecessary for users. In our study, we evaluated the instruction leaflets to represent 890 g per procedure (∼0.8 kg of CO_2_-e). It should not be neglected, regarding the annual number of procedures. It is thus of high relevance to change the Medical Device Regulation, giving industrials an obligation to provide the Instructions for Use in a non-paper format (e.g. a QR code).

### STEP 4. Recycling the majority of devices

Most parts of the devices used during the AF ablation procedure are made from polymers. These materials are incredibly cost-effective to produce and lightweight to transport when compared with their metal and glass predecessors. They offer many benefits such as sterility, strength, durability, and safety. Unfortunately, the counterpart is the generation of large amount of waste because of single use. Healthcare facilities in the USA generate ∼14 000 tons of waste per day, most of which is being disposed of in landfills or by incineration.^21^

As previously discussed, most of the polymers used have the potential to be recycled. Today, a paradigm shift is occurring in the polymer healthcare landscape as traditional linear models of resource consumption are being changed in favour of more circular approaches.^18^ It would be possible to further reduce the carbon footprint of the AF ablation procedures by recycling devices, and not only their packaging. However, recycling is possible only if polymers can be easily dismantled from medical tools, which is often not the case with current devices. Eventually, the simulations reported by Joseph *et al*.^18^ indicated the possibility of reducing 7.8 kg of CO_2_-e per procedure. It is important to consider with precaution these data which depend strongly on the scenarios considered.^22^

So far, our propositions require adaptations and the implementation of dedicated procedures, but not a complete restructuring of the sector. The next proposals need deeper changes but would further improve the carbon footprint.

### STEP 5. Redesigning some medical devices using eco-design approach

This proposition is based on the principle of eco-design for medical devices,^23^ which change the paradigm of development as described in the work of Arun Kumar.^24^ This redesigning phase applied to the healthcare sector following an ecological approach is an interesting solution to improve the carbon footprint while guaranteeing patient safety.^25^

In the case of the AF ablation procedure, the patches used for mapping are a good example of product that can be optimized. For each procedure, six mapping patches combined with six cables are used, and as a result, an important part of the carbon footprint comes from the single-use electrical cables, i.e. 41%. Accordingly, building a structure with reusable cables and disposable electrodes (like a cardiac monitor) could lead to a reduction of 2.8 kg of CO_2_ per procedure.

Another finding was the high-quality polymer used for building the handle of catheters that is usually uncommon for single-use material. Reducing material quality, in favour of recyclable and ecofriendly materials, could be a simple way to save energy while ensuring patient safety.

### STEP 6. Reusing some medical devices

The regulation of the European Union (regulation 2017/745) allows the ‘reprocessing’ which refers to a process carried out on a used device to allow its safe reuse. In general, it includes cleaning, disinfection, sterilization, and related procedures, as well as testing and restoring the technical and functional safety of the used device.^26^ According to Article 17 of medical device regulation, reprocessing of single used device is possible only if permitted by national law, which is not the case in most Western countries.

However, a recent study showed that most of the physicians agree to use reprocessing materials.^27^ Schulte *et al*.^22^ proposed a framework to help the reprocessing of catheter used in an AF ablation procedure. In view of the current design of diagnosis catheters, we estimated that it could be possible to reuse them four times without technical properties degradation. This assumption needs to be validated by a dedicate study. The goal here is to illustrate the possible benefit of reprocessing to reduce carbon footprint. The simulation based on the scenario proposed by Schulte *et al*.^22^ has pointed out that the four-times used materials leads to a decrease of 5.8 kg of CO_2_-e per procedure. This result should be put in balance with the analysis reported on Leiden *et al*.^12^ to figure out whether the disposal system or the reusable one leads to lower environmental impact. The reprocessing technique clearly paves a new way for the development in healthcare market, but further studies need to be thoroughly investigated to convincingly confirm its efficiency and reliability.^28^

The different proposals discussed above could lead to a total diminution of 23.7 kg of CO_2_-e per procedure (i.e. 30.8% of total carbon footprint). Considering a centre performing 500 procedures per year, 12 tons of CO_2_-e would be saved annually, which is equivalent to 65 000 km of car ride (*Figure 7*).

### Study limitations

Our study was limited to the material used in the operating room and the preoperative and post-operative phases of hospitalization were not considered. The ecological impact of indirect emissions was not evaluated. It includes building construction, equipment depreciation over years (computers, screens, ultrasound scanner…), transportation of personal and patients, among others. Finally, some data may have been omitted, and approximations were needed for missing data especially concerning the modes of transport of material and products. Ablation catheters used in this study were only coming from two companies (Biosense Webster and Medtronic), but our results should be reproducible with other partners as equipment are likely made in a similar way.

### Perspectives

There is a slight awareness concerning ecological impact of health care system in the global warming effect. Incentive programmes already exist for other sectors like food-waste and energy. However, there is no quantification of healthcare GHG emissions.

While individual and local awareness and initiatives are important, major changes should be achieved on a large scale, through national and international incentives. This study claims to sensitize the cardiologists, and more broadly the healthcare actors of this field, to generate an environmental electroshock in the cardiac EP community.

*Table 2* lists some ideas of actions needed to reduce the environmental impact of AF catheter ablation.

**Table 2:** Actions needed to reduce environmental impact of AF catheter ablation

Industrials	Physicians	Institutions	Legislation
Optimizing packaging	Be aware of the carbon impact when using materials	Improving recycling and sterilization processes	Inciting industrial to recycle their products
Removing ‘instruction for use’ in paper format	Choosing brand with environmental criteria	Considering carbon footprint in response to a call for tender	Promoting research in ecological field
Enhancing material conception	Creating task forces and group of experts to work on eco-audit tools	Standardizing a method of eco-audit that can applied for any product	Alleviating unnecessary and restrictive rules
Limiting non-re-usable, and non-recyclable materials.	Identifying the most polluting device and publishing data to inform the medical community		Implementing disposable system in hospitals
Developing re-usable, recyclable, and biodegradable materials			Allowing reuse of medical devices after reprocessing
Displaying a carbon footprint estimation for each product			

Working together and exchanging information to understand the needs of surgeons as well as the technical locks of industries. These actions are necessary for researchers to get involve in the development of new process and materials that are more friendly environmental.

## Conclusions

Atrial fibrillation catheter ablation and interventional cardiology in general is a significant contributor to GHG emissions and climate change. With an eco-audit method, we determined each AF catheter ablation produces 76.9 kg of CO_2_-e. Most of the carbon footprint is generated by EP material and especially catheters and patches. With the objective of the sustainability of our practice, we must rethink all the processes of single-use devices life. Modifications should integrate eco-designing, packaging modifications, recycling, and reusing. Only the involvement of all actors (industrials, physicians, institutions, and regulatory agencies) will allow concrete and durable actions.

## Data Availability

The data underlying this article will be shared on reasonable request to the corresponding author.
